# Case report: A case of metastatic BRAFV600-mutated melanoma with heart failure treated with immune checkpoint inhibitors and BRAF/MEK inhibitors

**DOI:** 10.3389/fonc.2024.1366532

**Published:** 2024-03-11

**Authors:** Aya Nishizawa, Misaki Kawakami, Yasuyuki Kitahara

**Affiliations:** ^1^Department of Dermatology, Tokyo Metropolitan Cancer and Infectious Diseases Center, Komagome Hospital, Tokyo, Japan; ^2^Department of Cardiology, Tokyo Metropolitan Cancer and Infectious Diseases Center, Komagome Hospital, Tokyo, Japan

**Keywords:** metastatic malignant melanoma, BRAFV600 mutation-positive melanoma, immune checkpoint inhibitor, BRAF/MEK inhibitors, heart failure, COVID-19 vaccination

## Abstract

**Background:**

Novel therapies, immune checkpoint inhibitors (ICIs), and BRAF/MEK inhibitors (BRAFi/MEKi) provide unprecedented survival benefits for patients with advanced melanoma. However, the management of drug-induced adverse events is problematic for both agents and, although rare, can cause serious cardiac dysfunction.

**Case report:**

A 42-year-old male patient with no significant medical history noticed a fading dark brown patch on his left anterior chest, which had been there for 20 years, after his second coronavirus disease 2019 (COVID-19) vaccination. The left axillary lymph node became swollen one week after a third booster vaccination. Thinking of it as an adverse reaction to the vaccine, but the swelling increased, so he visited a hospital. The patient presented with a brown macule with depigmentation on the left anterior chest and a 13 cm left axillary mass. A biopsy of the axillary mass showed a metastatic malignant melanoma. Positron emission tomography (PET) showed an accumulation only in the axillary lymph nodes. One month after the initial diagnosis, the axillary mass had further enlarged. In addition, pleural effusion, ascites, difficulty breathing, and systemic edema appeared, and he was diagnosed with heart failure (NYHA class III). Echocardiography showed an ejection fraction of 52% and electrocardiogram (ECG) showed no abnormal findings. Though it was (a life-threatening instead of the life-threatening) the life-threatening condition, we determined that the symptoms were associated with the current disease. Then nivolumab (nivo) plus ipilimumab (ipi) was initiated after explaining the risk of cardiac dysfunction associated with drug use to the patient. After initiation of ICIs, treatment was switched to BRAFi/MEKi (encorafenib/vinimetinib) after the patient tested positive for BRAF V600E. After one month of treatment, the tumor shrank significantly and achieved a complete remission after four months. Furthermore, as the tumor shrank, the patient’s heart failure improved, and he was able to continue treatment without serious drug-induced cardiotoxicity.

**Conclusion:**

Both ICI and BRAFi/MEKi carry a risk of cardiac dysfunction. However, without any underlying cardiac disease or severe cardiac dysfunction, their administration should not necessarily be excluded if careful follow-up is provided.

## Introduction

The prognosis of patients with malignant melanoma has improved significantly since the introduction of immune checkpoint inhibitors (ICIs) and tyrosine kinase inhibitors targeting *BRAF* mutations (1). In BRAF-mutant metastatic melanoma, both ICI therapy and molecularly targeted BRAF/MEK inhibitors (BRAFi/MEKi) are recommended as the first-line treatment. While these agents are likely to produce a high response rate, drug-specific adverse events can occur; ICIs carry a low but serious risk of myocarditis (2–5), and BRAFi/MEKi carry a risk of cardiovascular adverse events, including reduced left ventricular ejection fraction (6–9). Therefore, cardiac function should be assessed before starting treatment with these drugs, and the indications for their use should be carefully evaluated. We report herein a case of metastatic malignant melanoma with heart failure in which ICIs and BRAFi/MEKi were administered without any serious adverse events and complete remission was achieved by using BRAi/MEKi as second-line therapy after ICI administration.

## Case presentation

**Figure 1:**
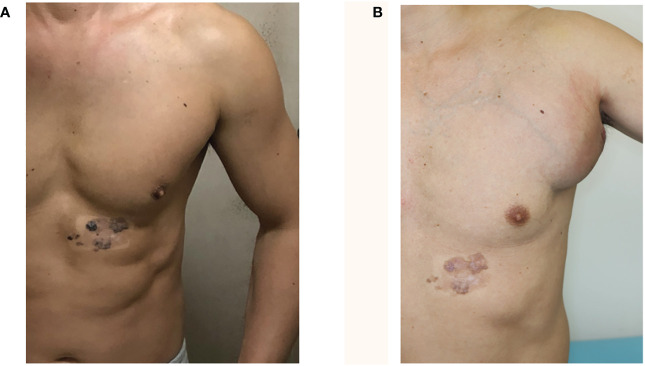
Clinical findings (pretreatment). **(A)** One month after second dose of COVID-19 vaccine (Sep. 2021). Following the second vaccination, the dark brown macule on the left anterior thoracic region, which had been present for 20 years, partially faded. **(B)** Seven months after the third dose of COVID-19 vaccine (Oct. 2022). The left axillary lymph node showed swelling one week after the third vaccination on the left arm. The swelling increased rapidly over several months. The dark brown macule on the left anterior chest spontaneously resolved. Edema was present in the trunk and extremities.

A 42-year-old, male patient with no remarkable medical history noticed that a dark brown macule on his left anterior chest, which he had been there for 20 years, began fading after his second coronavirus disease 2019 (COVID-19) vaccination. One week after he received his third booster vaccination, his left axillary lymph node became swollen. Thinking that it was an adverse reaction to the vaccine, the patient did not seek any treatment. However, the swelling increased rapidly over a few months, prompting him to visit our hospital (**Figure 1**).

**Figure 2:**
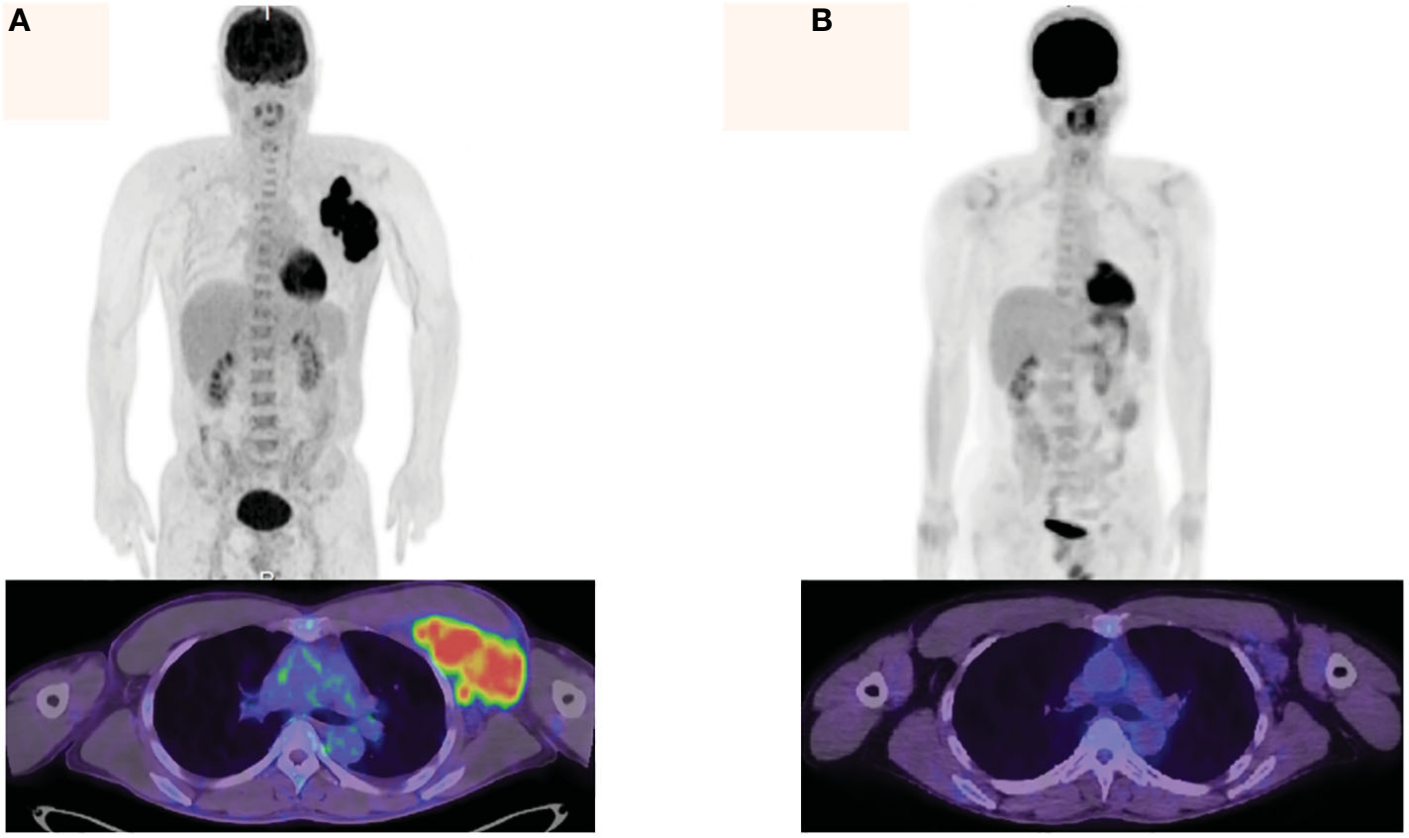
PET findings. **(A)** Before the start of the treatment. Accumulation was observed in the axillary lymph nodes (SUV max 13.2), but no metastasis to the other organs or accumulation in the primary tumor was observed (left thoracic macule). **(B)** Six months after encorafenib/binimetinib therapy. No accumulation was found at any site. Positron emission tomography, PET.

The patient had an indistinct, light-brown macule on the left anterior chest and a 13-cm left axillary mass with poor mobility. A biopsy specimen of the macule demonstrated an aggregation of melanophages only in the dermis and an absence of tumor cells. However, a biopsy of the axillary mass revealed metastatic melanoma. Based on these findings, an axillary lymph node metastasis of malignant melanoma originating in the left anterior chest lesion was diagnosed. Positron emission tomography (PET) demonstrated axillary lymph node accumulations (SUV max 13.2) with no metastasis to any other organ or accumulation in the primary tumor (the brown macule) (**Figure 2A**). Within one month after the initial visit, the axillary mass grew to 17 cm. Pleural effusion, ascites, and systemic edema were also present. The SpO2 had decreased to 88% on room air, and dyspnea, hypertension (blood pressure 158/111), tachycardia (heart rate 136 bpm), and congestive heart failure were also observed. Blood analysis demonstrated an elevated white blood cell count (11400/µL), hypoalbuminemia (Alb 2.2 g/dl), mildly elevated lactose dehydrogenase (LDH) (366 IU/L), elevated C-reactive protein (CRP) (5.64 mg/dl), and elevated N-terminal fragment-pro B-type natriuretic peptide (NT-proBNP) (766U/L). Cytology of the pleural fluid returned negative for tumor cells, and echocardiography demonstrated an ejection fraction of 52% and mildly reduced left ventricular contractility. An electrocardiogram (ECG) demonstrated a normal ST segment and sinus rhythm and no pulse irregularities or QT prolongation.

?>The patient was admitted on an emergency basis for his life-threatening condition. Treatment with a combination of nivolumab (nivo) plus ipilimumab (ipi) was begun after the associated risk of cardiac dysfunction was explained to the patient prior to testing for BRAF mutations. Simultaneously, he received diuretics and antihypertensive drugs for his heart failure. The axillary tumor shrank to 11 cm after three courses of the therapy, resulting in a partial response (PR). Grade 3 anemia and grade 2 fever occurred as immune-related adverse events. However, no other, serious adverse events, including myocarditis, were observed, and the patient was able to continue treatment.

**Figure 3:**
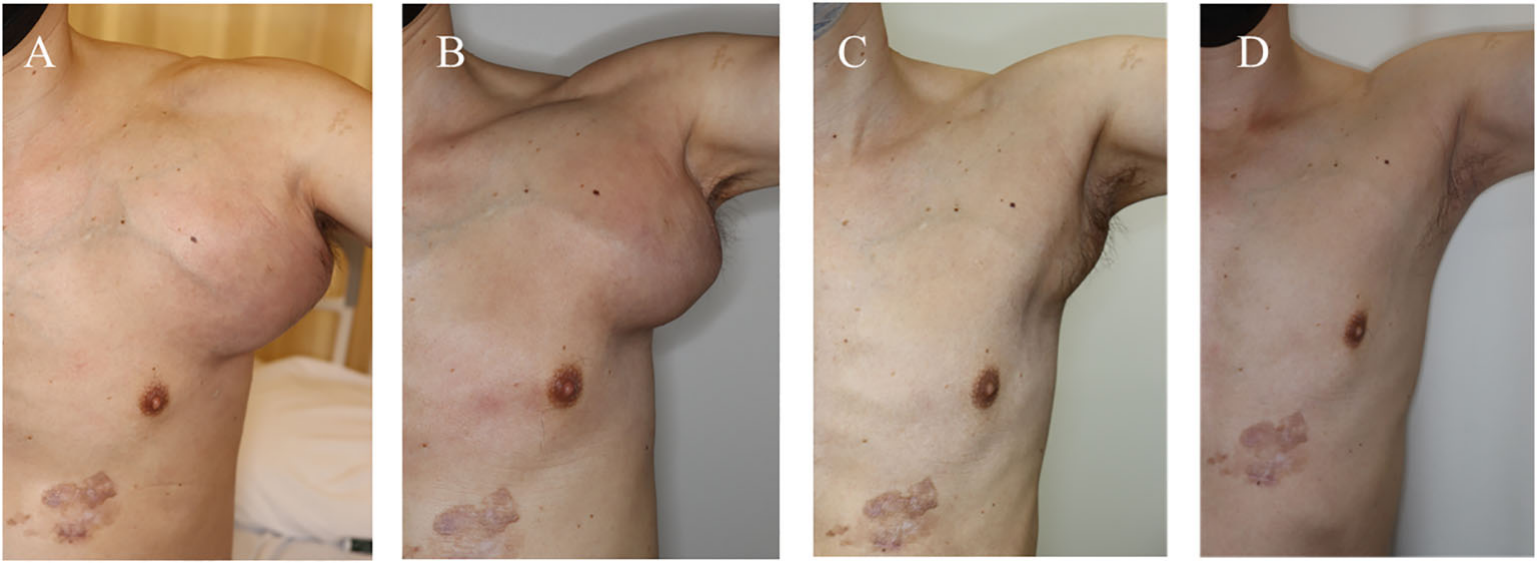
Clinical progress. **(A)** Before the start of nivo plus ipi therapy. The axillary mass increased in size to 17×18cm; **(B)** After 4 courses of nivo plus ipi. The axillary tumor shrank to 13×11cm. **(C)** After 1 month of encorafenib/binimetinib. The axillary mass shrank markedly to 6 x 8 cm.; **(D)** After 4 months of encorafenib/binimetinib. The axillary mass was no longer palpable. Nivo, nivolumab; ipi, ipilimumab.

**Figure 4:**
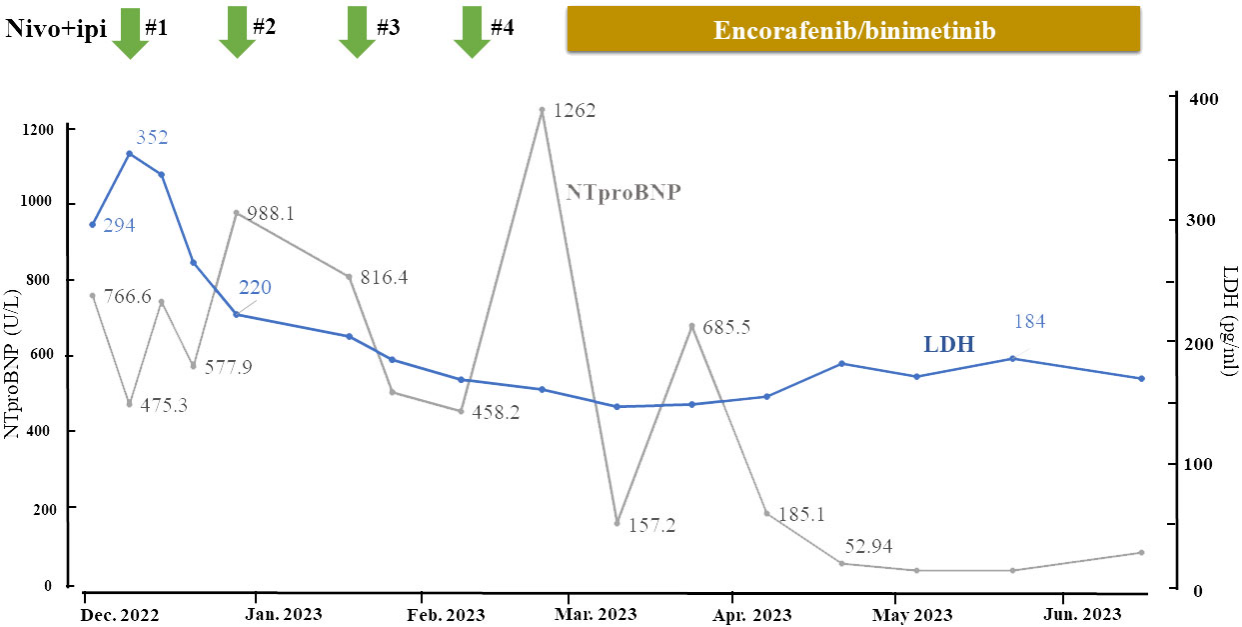
Clinical course. Change in serum LDH and NTproBNP during ICI and BRAFi/MEKi therapy. Serum LDH was elevated before the start of ICI therapy but normalized after two courses. NTproBNP initially decreased after the start of ICI therapy but began increasing after four courses, prompting a switch to BRAFi/MEKi therapy. The NTproBNP value then decreased along with shrinkage of the axillary mass and normalized within two months.

Unfortunately, after four courses of the therapy, the axillary tumor began growing. In addition, the heart failure flared, and new, mediastinal lymph node metastases appeared. Progressive disease (PD) was diagnosed, and the treatment was switched to BRAFi/MEKi (encorafenib/vinimetinib) after the patient tested positive for BRAF V600E. The tumor shrank markedly, and the heart failure improved after one month of this treatment (**Figures 3**, **4**).

At month 4 after the start of the BRAFi/MEKi therapy, the patient achieved complete remission (CR), and at month 6, no PET accumulation was found at any site (**Figure 2B**). Although grade 3 hypertension was observed as a drug-related adverse event, it was controlled with antihypertensive medication. No other serious adverse events, including cardiovascular adverse events, were observed. The patient has continued to receive BRAFi/MEKi and has maintained CR for more than six months.

## Discussion

The present case demonstrated that ICIs and BRAFi/MEKi can be safely administered to patients with BRAF V600E mutation-positive, unresectable metastatic malignant melanoma with heart failure and that the treatment was highly effective as second-line therapy following ICI therapy. However, to the best of our knowledge, there are no cases of ICI or BRAF/MEKi therapy associated with a risk of cardiac dysfunction being administered to patients with a malignant tumor and heart failure in which the treatment was successful or had no serious adverse effects.

Systemic therapy is always indicated for unresectable metastatic melanoma. In particular, immunotherapy with programmed cell death protein 1 (PD-1) antibodies (nivolumab, pembrolizumab) alone or in combination with cytotoxic T lymphocyte-associated antigen-4 (CTLA-4) antibodies (nivolumab plus ipilimumab) may be considered as the first-line treatment for BRAF wild-type melanoma (10). In patients with BRAF-V600 E/K mutations, first-line treatment with BRAF/MEK inhibitors can serve as an alternative to immunotherapy. In patients with BRAF-V600 E/K mutations with primary resistance to immunotherapy, treatment with a BRAFi/MEKi is recommended as second-line therapy (10).

Both ICIs and BRAFi/MEKi are thought to have greater therapeutic efficacy when combined than when used alone. The response rate for anti-PD-1 antibody monotherapy is around 40% (11, 12) whereas a higher response rate of 58% has been reported in patients receiving a combination of nivo plus ipi (13). Previously untreated patients and those with disease progression following ICI administration had a response rate of 63% to BRAFi/MEKi therapy (encorafenib/binimetinib) (14). Although this combination has demonstrated evidence of improved efficacy conferring a greater survival benefit, it was associated with a considerably higher incidence of treatment-related adverse events. The incidence of grade 3-4 immune-related adverse events (irAEs) potentially affecting all organs is reportedly 60% for nivo plus ipi (13). Although cardiovascular events have a low incidence of about 1%, they have recently begun receiving attention owing to a high, associated risk of mortality (2, 3). Specific cardiovascular events, such as myocarditis, pericarditis, acute coronary syndromes, and valvular heart disease, which can also be induced by ICI, can be induced by ICI and have an associated mortality rate of 50% (4, 5).

Cardiovascular adverse events may also occur with BRAFi/MEKi use. Phase III clinical trials of BRAFi/MEKi have demonstrated a LVEF reduction and hypertension onset in 10-20%, and QT interval prolongation in about 5%, of the subjects irrespective of grade. (8, 9)Therefore, cardiac function assessment is recommended for an early diagnosis and management of adverse events before starting ICI or BRAFi/MEKi therapy. As baseline, systemic assessment should include history-taking, a clinical examination, ECG, echocardiography, and a troponin T/I assessment (15). Our patient had heart failure with hypertension, low EF, tachycardia, and pleural effusion prior to initiating his treatment, and his heart failure developed after enlargement of the axillary mass.

Because the pleural fluid was negative for tumor cells and no metastatic lesions were found in the heart, the patient’s heart failure was considered to be related to the underlying malignancy rather than to a cardiac parenchymal issue. Due to the life-threatening nature of his condition, the patient was first treated for the malignant melanoma with ICI and BRAFi/MEKi, both of which were safely administered without any serious cardiotoxicity. Moreover, the heart failure improved as the tumor shrank. The fact that the metastases were confined to the lymph nodes and had not spread to the heart was key to the safe use of these drugs.

ICIs and BRAFi/MEKi are both first-line treatment options for patients with BRAF-mutated malignant melanoma. However, the crucial question of which treatment sequence is the more effective remains unknown. Two randomized trials, SECOMBIT and DREAMseq, are currently underway to investigate the optimal form of primary treatment in patients with BRAF-mutated unresectable melanoma (15–17). Our patient received ICIs as the first line treatment and following the failure of the initial treatment switched to BRAFi/MEKi, which elicited a good response as the second-line treatment.

It is also noteworthy that in the present case, the primary lesion spontaneously resolved, and the axillary lymph nodes rapidly swelled, after the patient received a COVID-19 booster vaccination.

The COVID-19 vaccine was rapidly developed and administered worldwide. Its most common side effects include injection site pain, fatigue, and muscle pain. Vaccination-associated axillary lymphadenopathy has also been reported in 7.86% of vaccine recipients (18). Therefore, it is important to determine whether axillary lymphadenopathy in a cancer patient is a vaccination-associated adverse effect or a lymphatic metastasis (19, 20).

There are a few, previous reports of the progression of lymph node metastases following vaccination (20, 21). In previous cases, a lymph node metastasis developed immediately after vaccination, suggesting an association with the vaccine. Malignant melanoma tends to regress spontaneously because of its strong immunogenicity. In the present case, the vaccine might have elicited a strong immune response, leading to the spontaneous resolution of the primary tumor while simultaneously promoting the axillary lymph node metastasis. The therapeutic effect of ICI therapy in the present case was initially thought to be PR but was eventually diagnosed as PD. Whether the strong immune response induced by the COVID-19 vaccine was synergistic or offset by the immune response induced by the ICIs remains unclear and should be discussed in more detail in future case series.

## Conclusion

ICI and BRAFi/MEKi therapy were safely administered to a patient with unresectable metastatic malignant melanoma with heart failure. There were no serious adverse events, and long-term tumor remission was achieved. In the absence of any underlying cardiac disease and severe cardiac dysfunction, the administration of these drugs should be considered if careful follow-up can be provided.

## Data availability statement

The original contributions presented in the study are included in the article/supplementary material. Further inquiries can be directed to the corresponding author.

## Ethics statement

Written informed consent was obtained from the individual(s), and minor(s)’ legal guardian/next of kin, for the publication of any potentially identifiable images or data included in this article.

## Author contributions

AN: Writing – original draft, Writing – review & editing. MK: Writing – review & editing. YK: Writing – review & editing.
